# *Pitx2c* Is Reactivated in the Failing Myocardium and Stimulates *Myf5* Expression in Cultured Cardiomyocytes

**DOI:** 10.1371/journal.pone.0090561

**Published:** 2014-03-04

**Authors:** Mario Torrado, Diego Franco, Francisco Hernández-Torres, María G. Crespo-Leiro, Carmen Iglesias-Gil, Alfonso Castro-Beiras, Alexander T. Mikhailov

**Affiliations:** 1 Institute of Health Sciences, University of La Coruña, La Coruña, Spain; 2 Department of Experimental Biology, University of Jaen, Jaen, Spain; 3 University Hospital Center of La Coruña, La Coruña, Spain; Tokai University, Japan

## Abstract

**Background:**

*Pitx2* (paired-like homeodomain 2 transcription factor) is crucial for heart development, but its role in heart failure (HF) remains uncertain. The present study lays the groundwork implicating *Pitx2* signalling in different modalities of HF.

**Methodology/Principal Findings:**

A variety of molecular, cell-based, biochemical, and immunochemical assays were used to evaluate: (1) *Pitx2c* expression in the porcine model of diastolic HF (DHF) and in patients with systolic HF (SHF) due to dilated and ischemic cardiomyopathy, and (2) molecular consequences of *Pitx2c* expression manipulation in cardiomyocytes *in vitro*. In pigs, the expression of *Pitx2c*, physiologically downregulated in the postnatal heart, is significantly re-activated in left ventricular (LV) failing myocardium which, in turn, is associated with increased expression of a restrictive set of *Pitx2* target genes. Among these, *Myf5* was identified as the top upregulated gene. *In vitro*, forced expression of *Pitx2c* in cardiomyocytes, but not in skeletal myoblasts, activates *Myf5* in dose-dependent manner. In addition, we demonstrate that the level of *Pitx2c* is upregulated in the LV-myocardium of SHF patients.

**Conclusions/Significance:**

The results provide previously unrecognized evidence that *Pitx2c* is similarly reactivated in postnatal/adult heart at distinct HF phenotypes and suggest that *Pitx2c* is involved, directly or indirectly, in the regulation of *Myf5* expression in cardiomyocytes.

## Introduction

The *Pitx2* homeobox transcription factor gene was originally identified as the candidate gene for the human Axenfeld-Rieger's syndrome [1], which is characterized by severe eye, teeth, craniofacial and umbilical abnormalities; less common features include heart defects [2], [3]. Shortly after its identification, *Pitx2* was found to play an important role in early development, as revealed by the generation of constitutive knockout mouse models. Consistent with *Pitx2* expression patterns, homozygous disruption of the mouse *Pitx2* gene led to mid-embryonic lethality due to defects in cardiac morphogenesis, in addition to severe abdominal wall and other tissue malformations [4]–[7]. Subsequently, the study of the *Pitx2* gene has become the object of continued research efforts aimed at identifying its role in the fetal, adult and diseased myocardium (reviewed in [8]–[11]).

Selective *Pitx2* deletion in the developing myocardium resulted in delayed differentiation of ventricular (but not atrial) cardiomyocytes, as development proceeded from embryonic to prenatal stages. During postnatal development, these mutants displayed dilatation and enlargement of right heart chambers and asymmetric hypertrophy of the interventricular septum associated with severely impaired ventricular systolic function [12]. Chamber-specific inactivation of *Pitx2* within atrial myocardium led to dilatation of both the left (LA) and right atrium (RA), in the absence of significant defects in ventricular chambers of mutant foetuses. However, after birth the mutants displayed a maladaptive remodelling of both atria and ventricles, associated with electrophysiological dysfunction, preferentially in the LA [13]. The atrial conduction system is particularly sensitive to *Pitx2* gene dose because mice heterozygous for *Pitx2* deficiency did not display altered cardiac morphology and contractile function in any of the heart chambers, but under electrical stimulation showed atrial arrhythmias [14], [15].

Three *Pitx2* transcript variants (*Pitx2a*, *Pitx2b*, and *Pitx2c*) have been identified in mammals, *Pitx2c* being the predominant or the only transcript detected in the adult mouse and human heart [13]–[17]. Of note, *Pitx2c* is differentially expressed across the cardiac chambers with maximal expression in the LA [15]. *Pitx2c* expression in the mouse LA is downregulated from fetal to postnatal stages [14]. The roles played by *Pitx2* within the four-chambered postnatal heart are poorly understood, even though its requirement is unquestioned. Conditional mouse mutants, in which *Pitx2* expression in atrial myocardium was turned off at birth, developed atrial ultrastructural remodelling and sinus node dysfunction [18]. *Pitx2* plays a pivotal role restricting pacemaker activity in the developing myocardium by repressing a nodal gene program and activating the working myocardium gene program [14], [19], [20]. Two groups independently found that *Pitx2c* expression is downregulated both in LA and in RA of patients with atrial fibrillation (AF), suggesting that *Pitx2c* dysfunction could be causatively linked to AF pathophysiology [13], [15]. Although using different *in-vivo* and *in-vitro* approaches, both groups provided congruent evidence that the reduction of *Pitx2c* expression in the adult atrial myocardium promotes its susceptibility to AF. These results are well in line with recent findings which suggest that *Pitx2c* loss-of-function mutations play a role in the genesis of the so-called “lone” AF in patients with structurally normal atria [21].

This study uncovers a novel, previously unrecognized link between heart failure (HF) and activation of the *Pitx2* gene in failing myocardium. To address this issue, we examined *Pitx2c* expression in left ventricular (LV) myocardium in the porcine model of diastolic HF (DHF) with preserved LV-ejection fraction. The expression of *Pitx2c*, physiologically downregulated in the postnatal heart, is significantly re-activated in failing myocardium which, in turn, is associated with increased expression of a restrictive set of *Pitx2* target genes. Among these, *Myf5* was identified as the top upregulated gene. *In vitro*, forced expression of *Pitx2c* in cardiomyocytes, but not in skeletal myoblasts, activates *Myf5* in dose-dependent manner. In addition, we demonstrate that the level of *Pitx2c* is upregulated in the LV-myocardium at systolic HF with severe reduced LV-ejection fraction in patients. Our present study provides the initial framework for analyzing putative contribution of *Pitx2c* re-activation in failing myocardium to the overall progression of HF, possibly through the involvement of *Pitx2c* in regulation of myogenic regulatory factor genes.

## Materials and Methods

### Heart failure porcine samples

Experimental procedures were carried out in accordance with the European Commission Directive 86/609/EEC on the protection of animals used for experimental and other scientific purposes, and all protocols were approved by the Animal Care and Use Ethical Committee of the University of La Coruña, Spain (approval N°:CE 012/2012). Newborn and early neonatal “Large White” piglets were obtained from a local commercial breeder (La Coruña, Spain), maintained in a conventional Nürtinger nursery system for days 6 after birth, randomized in two groups, and assigned to receive a single intravenous injection of isotonic PBS (phosphate-buffered saline) or 1.5–2.0 mg/kg of doxorubicin (Dox; Sigma, Madrid, Spain) [22], [23]. Untreated control group consisted of six non-injected piglets. On day 24 after injection (i.e., on day 30 after birth), hemodynamic parameters (surface ECG, blood pressure, heart rhythm, cardiac output, and extravascular lung water) were monitored in closed-chest piglets, whereas the measurements of LV end-systolic and end-diastolic pressure were performed in open-chest piglets as described [24], [25]. Dox-injected piglets displayed diastolic dysfunction (see Table S1). The piglets were euthanized and the entire heart was rapidly removed, weighed, and photographed while still beating. Then the isolated heart was placed on an ice-cold petri dish, partially sectioned at the midpoint of the LV length and photographs of the open ventricular chambers were taken. Immediately after this step, the LV free wall (LVFW) and left atrium (LA) were dissected into several samples (100–200 mg each). Samples were frozen in liquid nitrogen immediately after isolation and then stored until use at −80°C. The thickness of the LVFW was measured using digitized photoimages of the ventricular-chamber cross-sections as described [22], [26].

### Heart failure patient samples

Human heart samples were obtained from the heart tissue bank set up by MGC-L at the University Hospital Center of La Coruña (La Coruña, Spain). Written informed consent was obtained from patients or their relatives, and the use of human cardiac tissues was approved by the Ethical Committee of Clinical Investigation of Galicia, Spain (approval N°: 2007/239). The investigation conforms to the principles outlined in the Declaration of Helsinki. RNA and protein expression were assayed in explanted hearts from end-stage heart failure (HF) transplantation patients with ischemic (ICM) or idiopathic dilated cardiomyopathy (DCM) and compared with samples obtained from non-failing donor hearts that did not meet criteria for transplantation. All HF patients (functionally classified according to the New York Heart Association criteria) manifested a severely reduced systolic function; the donor group was not known to have any history of overt cardiovascular disease (see Table S2). Transmural samples biopsied from the anterolateral wall of the left ventricle (LV) of failing and non-failing human hearts were stored at −80°C until assayed for RT-PCR and Western blotting.

### RNA extraction and reverse transcription

Deep-frozen samples of piglet and human myocardium were directly disrupted in RLT buffer (Qiagen, Madrid, Spain) using a high-speed rotor-stator homogenizer (Ultra-Turrax T8, Germany), digested with Proteinase K (Qiagen), loaded onto RNeasy Mini/Midi columns (Qiagen), subjected to on-column digestion of DNA with RNase-free DNase (Qiagen), and processed in accordance with the manufacturer's recommendations. Total RNA from cultured HL-1 and Sol8 cells was extracted with the TriPure isolation reagent (Roche Diagnostics, Barcelona, Spain), treated with RNase-free DNase (Roche) for 1 h at 37°C and purified using a standard phenol–chloroform protocol. RNA yield and purity was determined spectrophotometrically, and RNA integrity was verified by running samples on 1.2% agarose gels and staining with ethidium bromide. Resulting RNA preparations were resolved in nuclease-free water (Ambion, Madrid, Spain) and kept at −80°C. Two microgram aliquots of individual total RNA were reverse transcribed using SuperScript III or SuperScript RNase H-minus (Invitrogen, Barcelona, Spain) reverse transcriptase and oligo-dT primers according to the manufacturer's instructions. Negative control reactions were performed in the same conditions without reverse transcriptase.

### Microarray

Total RNAs isolated from LV biopsies of three failing (i.e., Dox-injected) and three non-failing (i.e., PBS-injected) piglets were transported on dry ice to the “KFB – Center of Excellence for Fluorescent Bioanalytics” (University of Regensburg, Regensburg, Germany) and the quality of RNAs was assessed by using gel electrophoresis and spectrophotometry running eukaryotic total RNA Nano Series II program. The qualified RNA samples were independently hybridized on the Affymetrix GeneChip Porcine Genome Array (Affymetrix, Santa Clara, USA). Sample processing, array hybridization, scanning, and quantification were performed at the Affymetrix Service Provider and Core Facility, “KFB – Center of Excellence for Fluorescent Bioanalytics” (University of Regensburg, Regensburg, Germany; www.kfb-regensburg.de) as described in the Affymetrix GeneChip Expression Analysis Technical Manual. Briefly, the Ambion MessageAmp Premier kit, starting with 150 ng total RNA, was used for labeling. Hybridization was followed standard Affymetrix protocol in Affymetrix GeneChip Hybridization Oven 640. All chips passed standard quality controls to eliminate scans with abnormal characteristics (high versus low affinity binding). The scanning and image acquisition were performed by using the Affymetrix GeneChip Scanner 3000 7G and core software GCOS v.1.4. Expression data generation was performed by using the Affymetrix GeneChip Operating Software (GCOS v. 1–4; MAS5 algorithm with default settings). A set of the algorithms implemented in the Affymetrix Microarray Suite Version 5.0 was used for statistical treatment of the data. Genes were considered as altered if the folds-change was at least 2.0 and adjusted p value ≤0.05. The complete microarray data is available at NCBI through GEO (Gene Expression Omnibus) accession number GSE30110 (http://www.ncbi.nlm.nih.gov/geo).

### Conventional PCR

Conventional PCR was performed in a Biometra II system (Gottingen, Germany) as described [24]. The piglet LV/LA cDNAs were used as templates to detect different *Pitx2* transcript variants using the primers indicated in Table S3. The plasmid DNAs encoding pig full-length *Pitx2a*, *Pitx2b* and *Pitx2c* were used as a positive reference control. All PCR setups, including no-RT and no template (NT) controls, were performed at least in duplicate. Each PCR sample was resolved on a 2% agarose gel, and PCR products were visualized with ethidium bromide staining and UV illumination. Band intensity was estimated by densitometry (VersaDoc 1000) and Quantity One software (Bio-Rad, Madrid, Spain). The PCR products were cloned and sequenced (Secugen, Madrid, Spain) to confirm their identity.

### Real-time quantitative PCR (qRT-PCR)

qRT-PCR was performed on Bio-Rad IQ5 instrument (Bio-Rad, Madrid, Spain) and MxPro Mx3005p PCR thermal cycler (Stratagene, Madrid, Spain) using, respectively, SYBR Green (Bio-Rad, Madrid, Spain) and DyNAmo HS SYBR Green (Thermo Scientific, Madrid, Spain) master mix as described previously [24], [27]. The primer pairs were located in different exons to rule out genomic DNA amplification. Each primer pair used yielded a single peak of dissociation on the melting curve and a single band with the expected size on PAGE gels. Identity of the PCR products was confirmed by sequencing. NT and non-RT RNA template reactions were used as negative controls. All PCR setups were performed at least in duplicate. Relative quantifications were calculated with the comparative ΔCt cycle method with normalization to the expression of housekeeping genes coding for ribosomal protein L19 (*Rpl19*), glyceraldehyde-3-phosphate dehydrogenase (*Gapdh*) and β-D-glucuronidase (*Gusb*). The efficiency of target and reference amplifications was tested to be approximately equal. Primer sequences and additional data are given in Table S3.

### Plasmid constructs

Pig *Pitx2a* (sequence deduced from the swine genomic sequence NW_003610943) and *Pitx2c* (NM_001206435) were amplified from oligo-dT-primed cDNA from left atrium of newborn piglets; pig *Pitx2b* (sequence deduced from the swine genomic sequence NW_003610943) was amplified from skeletal muscle cDNA of 20-day-old neonatal animals. Each of the full-length *Pitx2* constructs was directionally cloned into p3xFLAG-CMV-14 expression vector (Sigma, Madrid, Spain) at the EcoRI (5′) and BamHI (3′) restriction sites and verified by sequencing [28]. Mouse full-length *Pitx2c* (NM_001042502.1) was amplified from Sol8 myoblast cDNA by PCR with specific primers containing HindIII and XbaI restriction sequences [27]. Subsequently, the PCR product was inserted into the pcDNA3.1/Zeo(-) plasmid (Invitrogen, Barcelona, Spain), which was modified to generate the V5-tagged *Pitx2c* as reported [29]. The plasmids were purified by using a PureLink HiPure plasmid filter purification kit (Invitrogen, Barcelona, Spain) according to the manufacturer's protocol. Purified plasmids were formulated in nuclease-free water (Ambion, Madrid, Spain).

### Cell culture and transfection in vitro

The following cell lines were used: COS-7 (a fibroblast-like cell line derived from monkey kidney tissue was purchased from the European Collection of Cell Cultures, Salisbury, Wiltshire, UK), HL-1 (a mouse atrial cardiac myocyte cell line was provided by Prof. William C. Claycomb [30]), and Sol8 (a mouse skeletal myogenic cell line was purchased from the American Type Culture Collection, Barcelona, Spain). COS-7 and Sol8 cells were cultured in Dulbecco's modified Eagle's Medium (Gibco, Barcelona, Spain) supplemented with 10% fetal bovine serum and penicillin–streptomycin–glutamine (Gibco); HL-1 cells were cultured in similarly supplemented Claycomb's growth medium [30]. Cells were trypsinized at 70–80% confluence and cell numbers were determined using an automated cell counter (Countess, Invitrogen, Barcelona, Spain). Cells were plated at a density of 10^5^ cells per 35 mm well, allowed to attach overnight and transfected with plasmids expressing: (1) FLAG-tagged pig *Pitx2a*, *Pitx2b* and *Pitx2c* (COS-7 cells/500 and 1000 ng of plasmid DNA), and (2) V5-tagged mouse *Pitx2c* (HL-1 and Sol8 cells/100–400 ng of plasmid DNA). All transfections were carried out with Lipofectamine LTX and PLUS Reagents (Invitrogen, Barcelona, Spain) following the manufacturer's instructions. For each plasmid, from three to six separate transfection assays were employed, and in each assay, transfections were performed in duplicate. The transfection protocol typically yielded 60–70% transfection efficiency, as revealed by transfection of CMV-EGFP (enhanced green fluorescent protein) vector [27]. Controls consisted of mock- and empty vector–transfected cells. For *Pitx2c*-silencing experiments, HL-1 and Sol8 cells were seeded in twenty four-well plates (30000 cells per well) and co-transfected with V5-tagged *Pitx2c* (at 400 ng) and *Pitx2c*-specific siRNA heteroduplex [13] (at 80 nM) using the Lipofectamine 2000 Transfection Reagent (Invitrogen, Barcelona, Spain), following the manufacturer's protocol. The cells transfected with V5-tagged *Pitx2c*, under the same conditions, were used as a reference. In each assay, transfections were performed in triplicate. The cells were harvested at 24–48 hours after transfection and processed for RNA and protein extraction as described [24], [27].

### Antibodies

**Figure 1:**
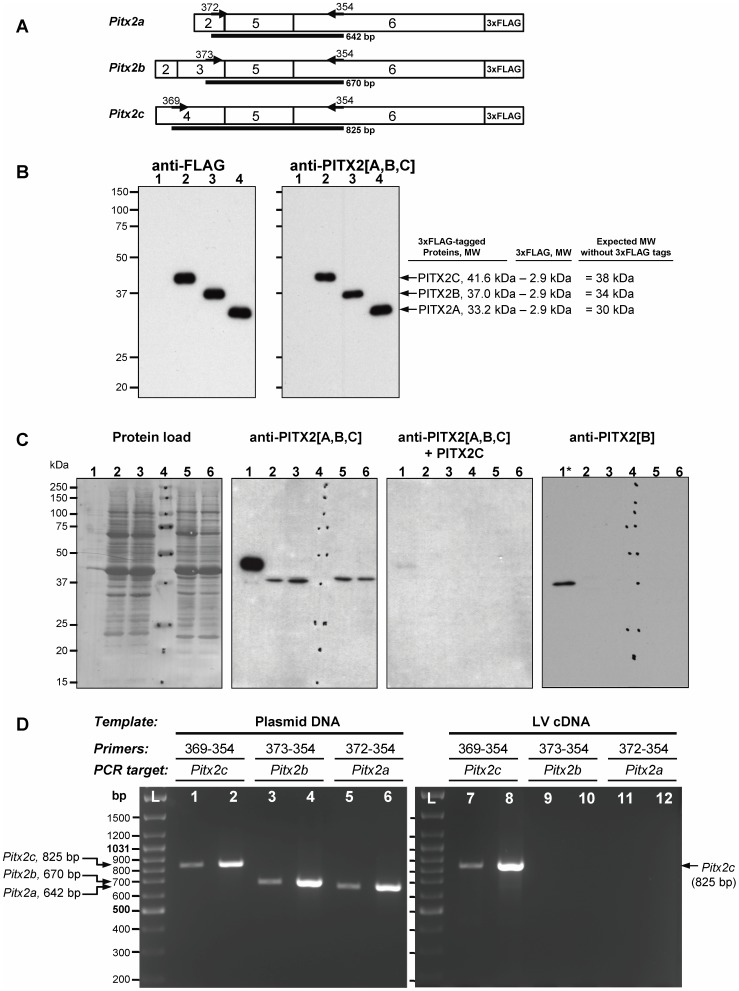
PITXC, but not PITX2A and PITX2B, is detected in the normal porcine and human left ventricular myocardium. **A -** Schematic representation of FLAG-tagged *Pitx2a*, *Pitx2b* and *Pitx2c* constructs. 2–6 – exons. The approximate location of the primers for downstream PCR analyses (**D**) is indicated. The expected size of the corresponding amplicons is shown in black lines. **B** - Lysates of COS-7 cells transfected with FLAG-tagged *Pitx2* constructs were electrophoresed and immunoblotted with anti-FLAG or anti-PITX2A,B,C antibodies. Cells transfected with: vector only (Lane 1), *Pitx2c* (Lane 2), *Pitx2b* (Lane 3) and *Pitx2a* (Lane 4) constructs. **C** - Lysates of COS-7 cells transfected with FLAG-tagged *Pitx2c* (Lane 1) or *Pitx2b* (Lane 1*) and left ventricle (LV) samples from normal 30-day-old porcine (Lane 2 and 3) and non-failing human (Lane 5, 6) hearts were blotted and probed with anti-PITX2A,B,C or anti-PITX2B antibodies. Lane 4 – Precision Plus WesternC Standards, kDa. Protein load – membrane stained with Amido Black 10B. Anti-PITX2A,B,C+PITX2C – antibodies neutralized by the extract of COS-7 cells transfected with FLAG-tagged *Pitx2c* construct. **D** - PCR amplifications of *Pitx2* transcript variants using the *Pitx2*-encoding plasmid DNAs (left panel, lane 1–6) or porcine LV cDNA (right panel, lane 7–12) as templates. Reactions were run in duplicate using 2-fold dilutions of both templates with each primer pairs. L – DNA size standards (GeneRuler DNA ladder mix; Fermentas).

The following primary antibodies were used: (1) rabbit polyclonal antibodies to PITX2A,B,C (Capra Science, Ängelholm, Sweden; at 1∶2000 dilution), (2) rabbit polyclonal antibodies to PITX2B (Capra Science, Ängelholm, Sweden; at 1∶1000 dilution).The specificity of the anti-PITX2 antibodies was independently validated in this study by Western blot analysis of COS-7 cells expressing each PITX2 isoform (see Fig 1 A, B), (3) rabbit monoclonal antibodies to MYF5 (Abcam, Cambridge, UK; at 1∶10000 dilution), (4) rabbit polyclonal antibodies to cardiac troponin I (Abcam, Cambridge, UK; at 1∶40000 dilution), (5) rabbit polyclonal antibodies to cardiac calsequestrin-2 (Abcam, Cambridge, UK; at 1∶10000 dilution), (6) rabbit polyclonal antibodies to ANKRD1 (at 1∶1000 dilution) were generated by Davids Biotechnologie (Regenburg, Germany) using the N-terminal region of pig ANKRD1 as immunogen [22], (7) mouse monoclonal anti-FLAG M2 antibody (Sigma, Madrid, Spain; at 1∶5000 dilution), (8) mouse monoclonal anti-V5 antibody (Sigma, Madrid, Spain, at 1∶5000 dilution), and (9) mouse monoclonal anti-GAPDH antibody (Sigma, Madrid, Spain, at 1∶10000 dilution). Secondary peroxidase conjugated anti-rabbit and anti-mouse IgG (Fab-specific) antibodies were purchased from Sigma (Madrid, Spain).

### SDS-PAGE and Western blotting

Tissue/cell samples were homogenized in standard 2x Laemmli buffer (Invitrogen, Barcelona, Spain) supplemented with complete protease inhibitor cocktail (Roche, Madrid, Spain) as previously described [24], [25]. Following centrifugation at 20000 g for 30 minutes, the concentration of supernatant proteins was analyzed using the Bio-Rad DC Protein Assay Kit (Bio-Rad, Hercules, USA) according to the manufacturer's protocol. The protein extracts were normalized to total protein concentration; the results of normalization were confirmed by SDS-PAGE and Coomassie staining before Western blotting analysis [25], [31]. Protein supernatants (loading range of 5–15 µg/run) were resolved on a 12% SDS-PAGE (Mini-Protean-III, Bio-Rad, Hercules, USA) and blotted onto PVDF-membranes (Hybond-P, Amersham Biosciences, Barcelona, Spain). Molecular weight (MW) standards (Precision Plus Protein WesternC Standards from Bio-Rad and SeeBlue Plus2 Pre-Stained Standard from Invitrogen) were included on each gel. Blots were probed with the antibodies indicated above and visualized by the Super-Signal West Pico chemiluminescent substrate (Pierce Biotechnology, Madrid, Spain). Equivalence of protein loading was confirmed by Amido-Black 10B (Merck, Barcelona, Spain) staining of blots after immunodetection. The blots were re-probed with anti-cardiac calsequestrin 2 and anti-GAPDH antibodies as additional control for loading. Quantification of Western blot signals was obtained by using a Bio-Rad GS800 calibrated densitometer with Quantity One software.

### Statistical Analysis

Values presented are expressed as mean ± S.E.M. All comparisons between groups were performed using an unpaired Student's *t* test. Differences were considered statistically significant for p value ≤0.05.

## Results

### PITX2C is the predominant splice-variant isoform in porcine and human left ventricular myocardium

PITX2 protein expression was studied by Western blot in left ventricular (LV) samples of normal porcine and non-failing human hearts. The FLAG-tagged constructs containing each of the three different PITX2 isoforms (Fig. 1 A) were used to evaluate the specificity of antibodies against PITX2A,B,C. Lysates from COS-7 cells transiently transfected with constructs were probed by Western blotting with anti-FLAG and anti-PITX2A,B,C antibodies (Fig. 1 B). Anti-FLAG detection revealed the expression of three PITX2 isoform-specific products, each migrating in SDS-PAGE as one band. The same protein products were also detected by anti-PITX2A,B,C antibodies indicating that these antibodies specifically recognize all PITX2 isoforms equally well (Fig. 1 B, right panel). However, only one band, with MW around 38–39 kDa corresponding to the expected size of PITX2C, could be seen in both porcine and human LV samples when probed with anti-PITX2A,B,C antibodies (Fig. 1 C). After neutralization of these antibodies with FLAG-tagged PITX2C protein, neither band was detected on the LV-derived blots. In addition, the antibodies against PITX2B isoform did not reveal any band in LV-extracts assayed under the same experimental conditions.

The results of Western blot assays were consistent with the expression levels of *Pitx2* variants determined by PCR analysis (Fig. 1 D). In control experiments, with the use of *Pitx2*-encoding plasmid DNAs as templates, each of three *Pitx2* transcript variants was amplified with comparable efficiency (Fig 1 D, left panel). However, only the largest *Pitx2c* variant was detected in LV samples using the same primer sets (Fig. 1 D, right panel). Taken together, the results strongly evidenced that PITX2C is the most abundant and predominant isoform in the LV of pigs and humans.

### Ventricular *Pitx2c* expression is downregulated after birth but markedly reactivated in piglet diastolic heart failure

**Figure 2:**
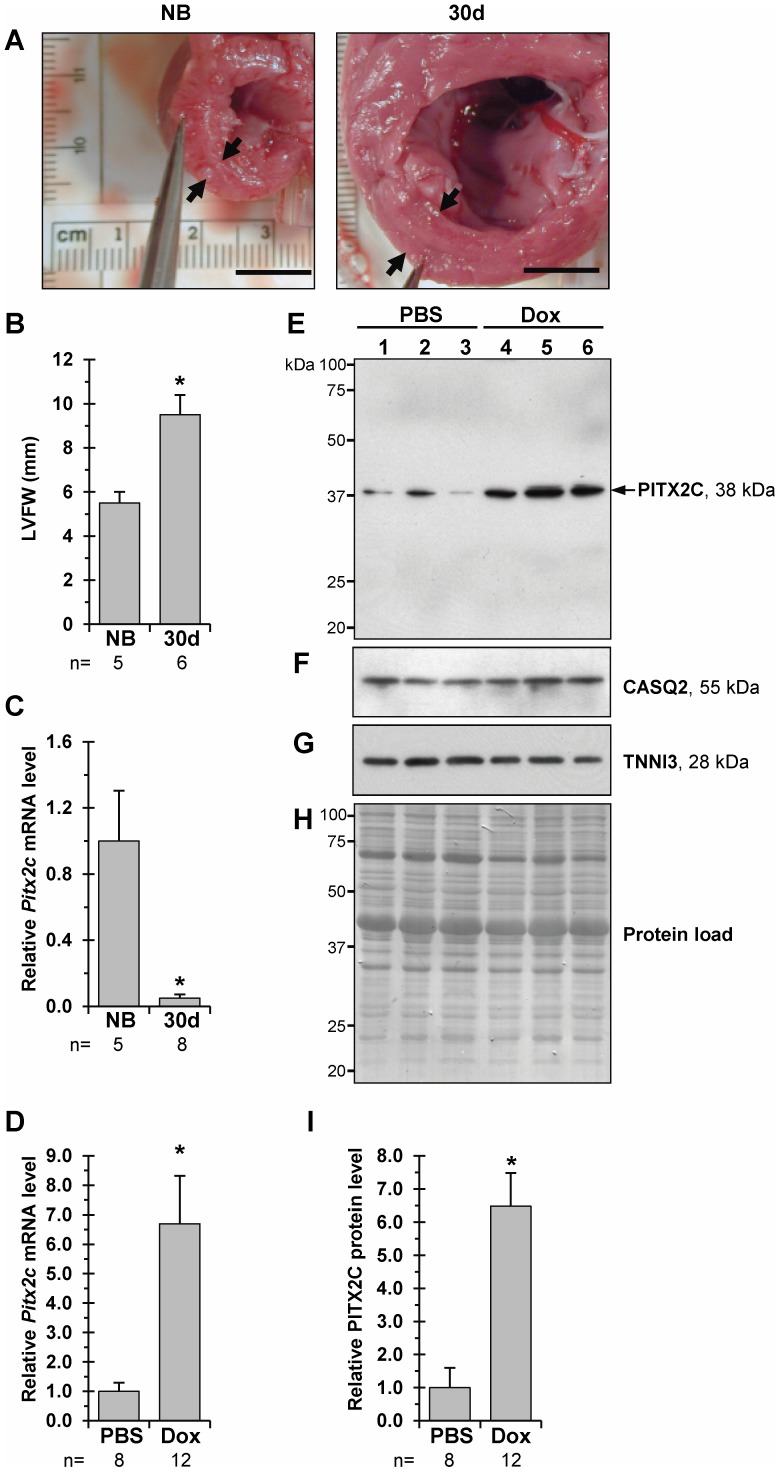
In the pig left ventricular myocardium, *Pitx2c* expression is downregulated after birth but re-activated in DHF. A, B - Cross-sections through left ventricular (LV) chambers of newborn (NB) and 30-day-old (30 d) piglets (Bar – 1 cm) and LVFW thickness values, respectively. C - Overall relative levels of *Pitx2c* transcript in LV-samples of newborn versus 30-day-old animals, *p≤0.05. D - Overall relative levels of *Pitx2c* transcript in LV-samples of PBS-injected versus Dox-injected 30-day-old piglets, *p≤0.05. E - Representative Western blots of PITX2C levels in the LV myocardium of PBS-injected (Lane 1, 2, 3) and Dox-injected (Lane 4, 5, 6) 30-day-old piglets. Each LV-sample (1–6) was derived from an individual animal. Western blot replicates were probed with antibodies against: (F) - cardiac calsequestrin 2 (CASQ2) and (G) - cardiac troponin I (TNNI3). MW values (kDa) of the bands detected are shown. H – Membrane stained with Amido Black 10B. I - Overall relative levels of PITX2C protein in LV-samples as based on average values from each group studied. *p≤0.05.

In neonatal piglets, the LV undergoes rapid hypertrophic growth as development proceeds [22], [32]. Likewise, in this work, we found that the average LVFW thickness was approximately 2-fold higher in normal 30-day-old piglets than in newborns (Fig. 2 A, B). Comparative qRT-PCR analysis of the LV-samples from these two groups of normal animals revealed that *Pitx2c* mRNA levels at birth are 16-fold higher than are those detected in 30-day-old animals (Fig. 2 C). These results suggest that a rapid progression of LV-concentric remodelling is associated with downregulation of *Pitx2c* expression in healthy neonatal piglets.

By utilizing our protocol [24], [25], diastolic heart failure (DHF) syndrome was induced in 6-day-old piglets by a single Dox injection. At day 30, neonatal piglets developed a diastolic dysfunction resulting in pulmonary congestion, but with nearly-normal (“preserved”) systolic function in terms of LV-end systolic pressure and global cardiac output values. The average fold-increase of both the normally spliced *Nppb* (natriuretic peptide precursor B) and *ΔE2-Nppb* (exon 2-skipped) mRNA [33] in the failing versus control LV myocardium was 5.8 and 9.4, respectively (see Table S1).

We quantified the relative amounts of the *Pitx2c* splice variant in the LV myocardium of control (i.e., PBS-injected) and experimental (i.e., Dox-injected) piglets (Fig. 2 D) by qRT-PCR. Expression of *Pitx2c* transcript was enhanced 6.7-fold in the failing versus control myocardium. The specificity of the unique *Pitx2c* amplification product was determined by melting curve and PAGE analyses (Figure S1 A–D). A similar 6.5 fold-increase was observed for the PITX2C protein in failing as compared to non-failing piglet LV myocardium (Fig. 2 E–I).

Thus, these results demonstrated that expression of *Pitx2c*, which is physiologically downregulated in the early postnatal heart, becomes significantly re-activated in an experimental DHF setting.

### *Myf5* is identified as the most upregulated gene among *Pitx2* targets in piglet failing myocardium

**Figure 3:**
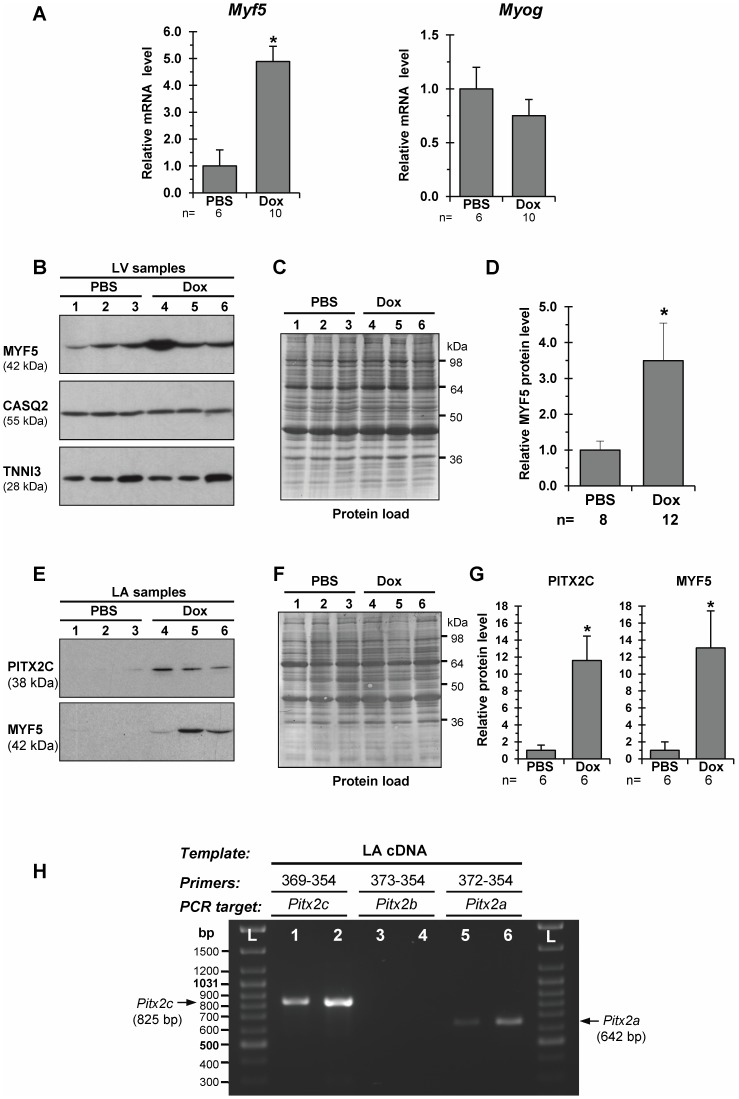
Expression of *Myf5*, but not *Myog*, is upregulated in the failing pig left heart as determined by qRT-PCR and Western blot analyses. A – Relative levels for *Myf5* (left) and *Myog* (right) transcripts in the left ventricular (LV) samples from PBS- and Dox-injected piglets. B - Representative Western blots of MYF5 levels in the LV samples from PBS- (Lane 1, 2, 3) and Dox-injected (Lane 4, 5, 6) piglets (blots probed with anti-PITX2A,B,C antibodies). Western blot replicates were probed with antibodies against cardiac calsequestrin 2 (CASQ2) and cardiac troponin I (TNNI3). MW values (kDa) of the bands detected are shown. C – Control of protein loading: Western blot membrane probed with anti-MYF5-antibodies was then stained with Amido Black 10B to detect blotted proteins. D – Overall relative levels of MYF5 protein in LV-samples as based on average values from each group studied. *p≤0.05. E – Representative Western blots of PITX2C and MYF5 levels in the left atrium (LA) from PBS- (Lane 1, 2, 3) and Dox-injected (Lane 4, 5,6) piglets. MW values (kDa) of the bands detected are shown. F – Membrane stained with Amido Black 10B. G – Overall relative levels of PITX2C and MYF5 in LA-samples as based on average values from each group studied. *p≤0.05. H – RT-PCR amplifications (Lane 1–6) of *Pitx2* transcript variants using the porcine LA cDNA as template. Reactions were run in duplicate using 2-fold template dilutions with each primer pairs. **L** – DNA size standards (GeneRuler DNA ladder mix; Fermentas).

Marked reactivation of PITX2C protein expression suggested that downstream targets could also be activated in DHF myocardium. A variety of putative targets of *Pitx2* have been suggested over the years, many in associative gene expression studies of non-cardiac tissues by using microarray-based methods [34]–[38]. In adult ventricular myocardium, the exact *Pitx2* signalling pathway is not defined, nor the *Pitx2* targets involved. Our microarray expression profiling of LV-samples from non-failed and failed porcine hearts revealed that *Pitx2* upregulation is associated with increased expression of a restrictive set of known *Pitx2* target genes (Table 1). It is noteworthy that previous studies have demonstrated that these genes have PITX2 binding site(s) in their promoter region, as demonstrated by chromatin immunoprecipitation and functional promoter-reporter assays (see references in Table 1). Changes in the expression of three transcription factors, *Myf5*, *Pax3*, and *FoxJ1*, as well as *Pitx2* itself were further verified by real time qRT-PCR in the same (three failing versus three non-failing) samples used in microarray assays (Table 1). Among *Pitx2* targets, most notable was the marked upregulation of the myogenic factor MYF5 in DHF myocardium. mRNA levels for the *Pax3* transcription factor, which can activate *Myf5* in embryonic muscle progenitor cells [39], were also elevated in failing LV-myocardium, although to a lesser extent as compared to *Pitx2c* and *Myf5* (see Table 1). *Pax3*, *Pitx2*, and *Myf5* are upstream genes which differentially regulate the kernel myogenic program in cell- and time-dependent manner [40], [41]. However, the expression of downstream myogenic factors - MYOG (myogenin; see Fig. 3 A, right panel), MYOD, and MYF6 (also known as MRF4) - was not significantly altered in failing compared to non-failing LV myocardium. In addition, there were subtle differences in the expression levels of skeletal muscle-specific genes (*Tnnt1*, *Tnnt3*, *Myh4*, and *Tpm2*), with exception of the *Myl1* gene which expression was markedly downregulated in failing myocardium (see Table S4 and complete microarray data set at GEO accession number GSE30110). LV expression of the *FoxJ1* (forkhead box J1) transcription factor, which is involved in cardiac morphogenesis and remodelling [42], was moderately upregulated in Dox-injected versus that in control PBS-injected animals (see Table 1).

**Table 1:** Upregulation of genes sensitive to Pitx2 dosage in the piglet DHF model.

Gene coding for	Gene symbol	Fold change		PITX2 binding to the promoter	Reference
		microarray	qRT-PCR		
Paired-like homeodomain 2	*Pitx2*	2.14	6.60±1.6*		
Natriuretic peptide precursor A	*Nppa*	4.42	HV	+	[73]
Cyclin D2	*Ccnd2*	2.38	HV	+	[51]
Forkhead box protein J1	*Foxj1*	2.66	3.20±0.4	+	[74]
Myogenic factor 5	*Myf5*	5.15	4.80±0.5	+	[57]
Paired box 3	*Pax3*	3.39	1.80±0.3	+	[45]

Three pairs of failing-non failing samples were assayed by both microarray and qRT-PCR. *Quantitative RT-PCR analysis with the use of the primer pair (350–353, see Table S3) annealing to the sequence common to the different *Pitx2* variants. HV – highly variable expression within each sample sets.

Recently, a search for cardiac *Pitx2* targets by ChIP-Seq and microarray assays revealed several novel putative targets negatively regulated by *Pitx2* in transfection assays using the 293FT cell line derived from human embryonic kidney [18]. The expression of these genes (coding of calcium voltage-gated channel subunit alpha-1d; potassium voltage-gated channel, KQT-like subfamily, member 1; caveolin 1; emerin) was not altered in LV myocardium of Dox-injected versus PBS-injected piglets (see complete microarray data set at GEO accession number GSE30110).

We further validated the upregulation of *Myf5* in DHF LV-myocardium by performing quantitative RT-PCR and Western blot analyses in a larger cohort of Dox- versus PBS-injected animals. On average, the level of *Myf5* transcript was enhanced 4.8-fold in the failing (n = 12) versus control (n = 8) LV-myocardium (Fig. 3 A, left panel). The specificity of the unique *Myf5* amplification product was determined by melting curve and PAGE analyses (see Figure S2). A seemingly similar 3.5 fold-increase was observed for the MYF5 protein in failing as compared to non-failing piglet LV myocardium (Fig. 3 B–D).

Recent clinical evaluations indicated that LV diastolic dysfunction can result in functional and structural remodelling of the left atrium (LA) in patients [43], [44]. In line with these data, we studied the expression of *Pitx2c* and *Myf5* in LA samples from Dox- versus PBS-injected piglets. As revealed by Western blot, the levels of both PITX2C and MYF5 were about 10-fold higher in the LA of Dox-treated as compared to those in the LA of PBS-treated piglets (Fig. 3 E–G). Only the 38-kDa band, corresponding to the porcine PITX2C isoform, was detected on LA-derived blots probed with anti-PITX2A,B,C antibodies (Fig. 3 E), although these antibodies, as we demonstrated, recognize the all PITX2 isoforms (see Fig. 1 B, right panel). The RT-PCR analysis for LA-samples (Fig. 3 H) revealed the predominant expression of the *Pitx2c* variant, while other *Pitx2* alternative transcripts were either not detected (*Pitx2b*) or detected in traces (*Pitx2a*).

Collectively, the findings from the porcine model of DHF revealed a strong upregulation of *Pitx2c* and *Myf5* under stress conditions, suggesting that *Pitx2c* could be involved in transcriptional regulation of *Myf5* expression, and probably to a lesser extent of *FoxJ1* too, in postnatal cardiomyocytes.

### Forced expression of *Pitx2c* increases the expression of *Myf5* in cultured cardiomyocytes

To explore the possibility that *Pitx2c* may activate *Myf5* and/or *FoxJ1* expression specifically in cardiomyocytes, we overexpressed the full-length *Pitx2c* variant in HL-1 cardiomyocyte as well as undifferentiated Sol8 skeletal muscle cell lines. The Sol8 cell line was used as a control since, as previously demonstrated, *Pitx2c* overexpression promotes proliferation and arrests muscle differentiation of Sol8 cells [45].

**Figure 4:**
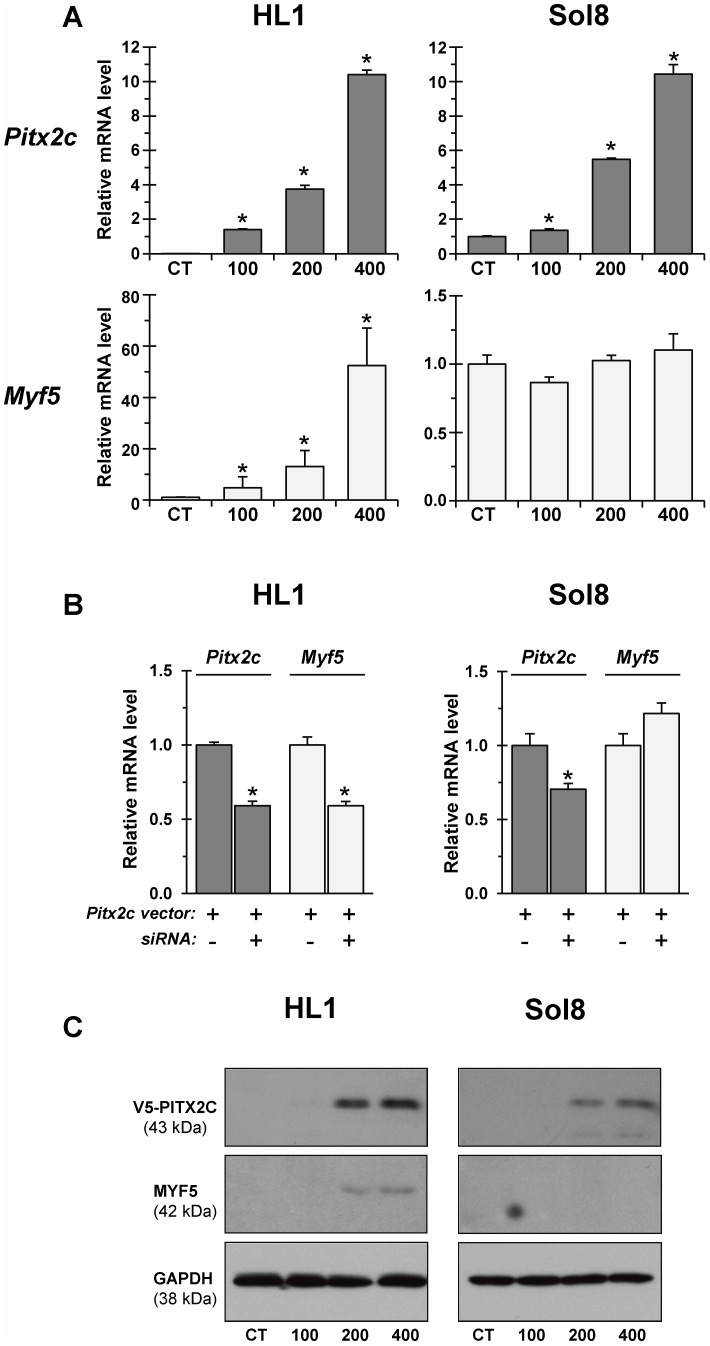
*In vitro* forced expression of *Pitx2c* results in increased *Myf5* expression in cultured cardiomyocytes but not skeletal myoblasts. A - Overall relative levels of *Pitx2c* (black) and *Myf5* (grey) transcripts in HL-1 and Sol8 cells transfected with V5-tagged *Pitx2c* vector at different doses (100-400 ng). CT – empty-vector transfected cells. Shown are results of qRT-PCR analysis. Data from six replicates of each transfection were pooled and averaged. In controls, the relative levels of transcripts for *Myf5* were assigned to a value of 1, whereas those for *Pitx2c* were assigned to a value of 10^−4^. *p≤0.05. B – Overall relative levels of *Pitx2c* and *Myf5* transcripts in HL-1 and Sol8 cells co-transfected with V5-tagged *Pitx2c* (at 400 ng) and *Pitx2c*-specific siRNA heteroduplex (at 80 nM). Data from three replicates were pooled and averaged. C - HL-1 and Sol8 cells transfected with V5-tagged *Pitx2c* (at 100–400 ng) were pooled (from triplicate wells in each transfections), lysed, electrophoresed, and immunoblotted with antibodies against PITX2A,B,C, MYF5 and GAPDH. MW values (kDa) of the bands detected are shown.

Transient transfections of HL-1 cardiomyocytes strongly enhanced, in a dose-dependent manner, the expression of the *Myf5* gene compared to that in mock- and empty vector-transfected cells. This effect appears to be cell-type dependent, as no stimulation of *Myf5* transcript expression was observed in *Pitx2c*-transfected Sol8 myoblasts (Fig. 4 A). In this direction, the inhibition of *Pitx2c* overexpression, using *Pitx2*-siRNAs, led to a decrease of *Pitx2c*-induced activation of the *Myf5* gene in HL-1 cardiomyoblasts, whereas co-transfection with *Pitx2c* siRNA did not affect the basal level of *Myf5* expression in *Pitx2c*-transfected Sol8 myoblasts (Fig. 4 B). The ability of *Pitx2c* to activate *Myf5* expression in HL-1 cardiomyoblasts was further confirmed by Western blot analysis. Ectopic PITX2C upregulation resulted in activation of MYF5 protein expression in HL-1, but not in Sol8 cells (Fig. 4 C). Under our experimental conditions, we could not detect any MYF5 protein in either control or *Pitx2c*-transfected Sol8 myoblasts.

With respect to expression of *FoxJ1*, the results of *Pitx2c* transfections of HL-1 cells were not very conclusive but there appeared to be a statistically significant and slightly dose-dependent increase of *FoxJ1* expression as compared to control cells (see Figure S3). By contrast, in transfected Sol8 myoblasts *FoxJ1* was downregulated at doses of 100 and 200 ng of *Pitx2c* plasmid DNA followed by a return to basal levels at a highest dose used (400 ng). The cause of this down-regulation of *FoxJ1* expression is not known at present.

We next asked whether forced expression of *Pitx2c* is able to modulate the expression of PAX3 and MYOG myogenic factors in HL-1 versus Sol8 cells. We found that forced *Pitx2c* expression does not affect the levels of *Pax3* expression in either HL-1 or Sol8 transfected cells, but results in decreased expression of the *Myog* gene selectively in Sol8 undifferentiated myoblasts (see Figure S3). The latter is in accordance with previous experimental data reporting *Myog* downregulation in Sol8 myoblasts transfected with *Pitx2c* expression vectors [27], [45].

Essentially, data from transfection assays showed that *Pitx2c* upregulation does result in strong and dose-dependent activation of *Myf5* expression in cardiomyocytes and to a minor extend of *FoxJ1*.

### Ventricular *Pitx2c* expression is reactivated in human systolic heart failure

**Figure 5:**
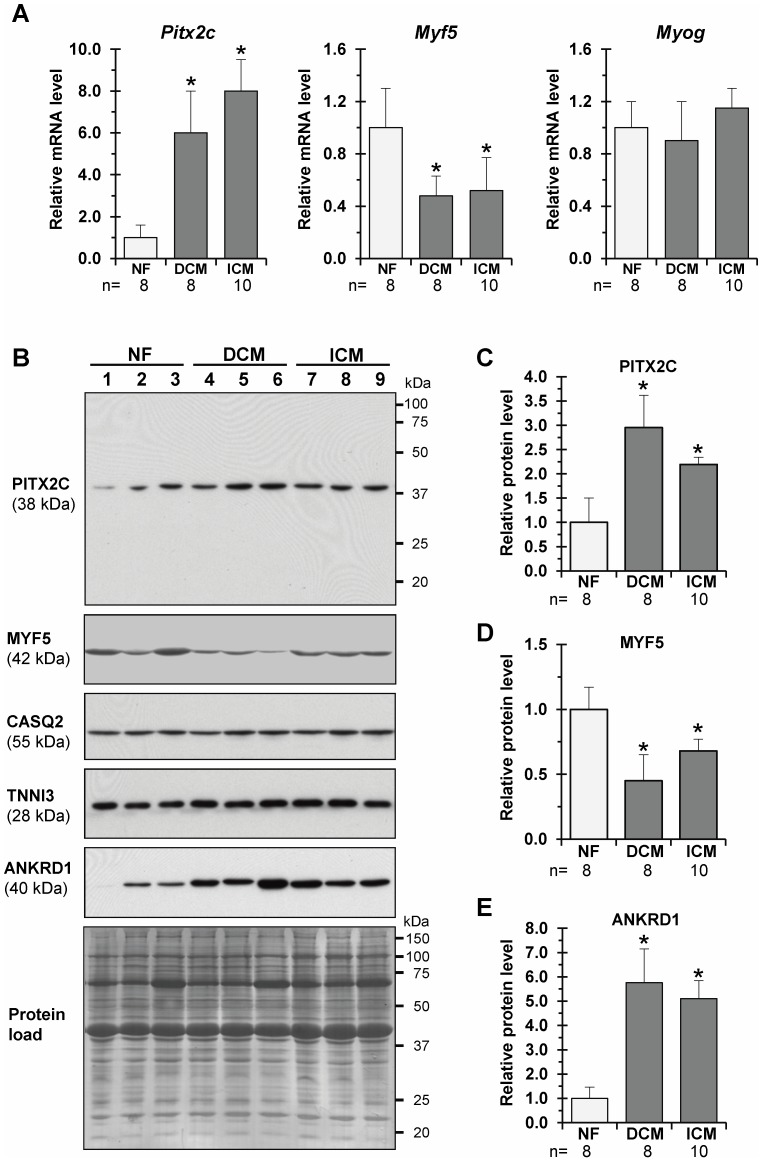
*Pitx2c* expression is reactivated, while expression of *Myf5* is downregulated in human systolic heart failure as determined by qRT-PCR and Western blot analyses. A - Overall relative levels of *Pitx2c*, *Myf5*, and *Myog* transcripts in the left ventricular (LV) samples from non-falling (NF) and failing human hearts due to idiopathic dilated (DCM) and ischemic (ICM) cardiomyopathy. *p≤0.05. B - Representative Western blots of PITX2C levels (top) in the LV samples from NF (Lane 1–3), DCM (lane 4–6) and ICM (lane 7–9) failing hearts. Western blot replicates were probed with antibodies against MYF5, cardiac calsequestrin 2 (CASQ2), cardiac troponin I (TNNI3) and ankyrin repeat domain 1 protein (ANKRD1). MW values (kDa) of the bands detected are shown. Protein load - Membrane stained with Amido Black 10B. Overall relative levels of PITX2C (C), MYF5 (D) and ANKRD1 (E) protein in LV-samples as based on average values from each group studied. *p≤0.05.

Controversy about whether diastolic (DHF) and systolic HF (SHF) represent overlapping [46] or distinct [47] phenotypes of severely impaired cardiac function remains unsolved. We aimed to investigate whether re-activation of *Pitx2c* expression in DHF, as revealed in the porcine model, is also related to SHF in patients undergoing heart transplantation due to dilated (DCM) or ischemic cardiomyopathy (ICM). On average, the level of expression of the *Pitx2c* transcript was nearly 6-fold (DCM) and 8-fold (ICM) higher in LV tissue samples from explanted hearts as compared with those from donor human hearts (Fig. 5 A). However, Western blotting analysis showed that PITX2C protein upregulation in falling human myocardium was significantly less (2–3-fold increase) than that observed at the RNA level (see Fig. 5 B, C). In the studied failing myocardium, the expression of ANKRD1 (ankyrin repeat domain 1 protein), the surrogate marker of HF [48], was significantly upregulated (Fig. 5 B, E), while the levels of TNNI3 (cardiac troponin I; the marker of acute myocardial ischemia) were unchanged or slightly augmented as compared to non-failing samples (see Fig. 5 B).

In addition, we studied the expression of *Myf5* and *Myog* genes in human failing hearts. Expression of *Myf5* was found to be nearly 2-fold decreased at both transcript (Fig. 5 A) and protein (Fig. 5 B, D) levels in falling as compared to non-failing human myocardium, whereas the expression of *Myog* was unchanged (Fig. 5 A). Although not statistically significant, there was a trend toward less decrease of MYF5 protein in ICM than in DCM samples (Fig. 5 D).

Collectively, the results suggest that *Pitx2c* re-activation in ventricular myocardium is a common molecular signature of HF with preserved (DHF) as well as reduced systolic (SHF) function, while the expression of *Myf5* is distinct between the two HF phenotypes studied.

## Discussion

In this paper we provide what are, to our knowledge, the first reported results, demonstrating: (1) the re-activation of *Pitx2c* expression in failing myocardium in different HF settings, (2) the marked upregulation of *Myf5* myogenic factor expression in failing ventricular and atrial myocardium as well as in cultured cardiomyocytes in response to forced expression of *Pitx2c*, and (3) the expression of the large repertoire of myogenic regulatory factors (MRFs) in normal and failing myocardium. Each of these data sets is discussed in the context of the underlying molecular mechanisms of HF.

### Activation of myocardial PITX2c expression as a hallmark of heart failure

A possible involvement of PITX2C in ventricular HF has not yet been explored. We demonstrate herein a similar upregulation of *Pitx2c* expression in the LV myocardium, at transcript and protein levels, in a porcine model of DHF as well as in patients with end-stage SHF. In patients, LV diastolic dysfunction can lead to elevation of LA pressure, resulting in increased LA wall tension and progressive LA dilatation, thereby increasing the risk and frequency of atrial fibrillation development [44], [49]. By using the porcine DHF model, we evidence that *Pitx2c* is upregulated in the LA myocardium. This new observation adds an additional layer of complexity to the challenge linking perturbations of *Pitx2c* expression in the LA to the development of atrial fibrillation [11]. Mechanistically, the results of this study strongly suggest that *Pitx2c* activation is a common feature of the failing heart, in line with recent reports of myocardial *Pitx2c* upregulation in HF due to ventricular septal defects in human foetuses [50]. The question is whether *Pitx2c* upregulation deteriorates the failing myocardium as one of the factors for HF progression or its activation is merely a secondary manifestation of impaired LV function that might even have a compensatory effect in HF? Certainly, any answer to such a question is still speculative as current knowledge on *Pitx2c* functions in postnatal ventricular myocardium is incomplete and largely uncertain. In addition, the role of *Pitx2c* in failing myocardium may be multifaceted depending on the cause, type, stage, and severity of HF. Our results in fact suggest that an excessive re-activation of *Pitx2c* expression could contribute to an overall disturbance of the cardiac gene regulatory network in failing myocardium.

*Pitx2* expression is upregulated by a canonical *Wnt*/β-catenin signalling pathway involved in activation of cell proliferation of embryonic cardiac precursors [51]. However, the canonical *Wnt* signalling negatively regulates the proliferation of adult cardiac progenitor cells, contributing to negative LV-remodelling [52]. There is substantial evidence that inhibition of the canonical *Wn*t/catenin pathway can be beneficial for HF [53]. Recently, *Pitx2* is recognized for being a downstream transcription factor in the TGF-β (transforming growth factor beta) signalling pathway in non-muscle cells [36]. Myocardial TGF-β signalling is activated in animal models of myocardial infarction and cardiac hypertrophy, and patients with dilated and hypertrophic cardiomyopathy. Reducing of TGF-β signalling is seen as a promising therapeutic approach for cardiovascular diseases (reviewed in [54], [55]). Notably, our microarray analysis revealed that the components of the TGF-β signalling pathway, such as TGFβR3 (TGFβ receptor 3; 6.2-fold increase) and SMAD3 (2.0-fold increase), are upregulated in the failing porcine LV-myocardium (see Table S4). Hence, it is likely that upregulation of the canonical TGF-β signalling pathway, that is believed to represent a maladaptive response of diseased heart to stress, may contribute to *Pitx2c* re-expression in porcine and human myocardium in advanced and late stages of HF. The endogenous targets of *Pitx2* in the adult heart remain largely enigmatic. Many genes involved in cell-junction assembly, ion transport and cell proliferation/migration were found to be upregulated in mouse mutants with conditionally inactivated *Pitx2* in the postnatal atrial myocardium, but no supporting molecular evidence of *Pitx2*-dependent downregulation of these genes in cardiomyocytes has been reported [18].

Although the data mentioned above do not directly involve *Pitx2c* signalling in pathogenesis of HF, our preliminary assumption is that *Pitx2c* re-activation in the failing heart might be maladaptive. The finding that forced expression of *Pitx2c* dramatically upregulates the myogenic transcription factor MYF5 in cultured cardiomyocytes, lends some credence to this suggestion.

### *Myf5* upregulation as a consequence of *Pitx2c* activation

Given that gene regulatory networks for cardiac and skeletal muscle are different in many ways, the enhanced expression of two key regulators of skeletal myogenesis - *Myf5* and *Pax3* - in failing porcine myocardium has attracted our special attention. To establish whether the upregulation of these genes is mediated via *Pitx2c*, HL-1 cardiomyocytes (with barely detectable *Myf5* transcript levels) and Sol8 skeletal myocytes (with low detectable *Myf5* transcript levels) were transiently transfected with *Pitx2c* expression vector. Forced expression of *Pitx2c* dramatically stimulated the expression of *Myf5* (at both transcript and protein levels) exclusively in HL-1 cardiomyocytes, suggesting such regulation is cell-content dependent. The observation that the expression of *Myf5* is decreased following *Pitx2c* silencing in only HL-1 cardiomyoblasts, overexpressing *Pitx2c*, does also argue in this direction. In *Pitx2c*-transfected HL-1 cardiomyocytes, augmented expression of both *Pitx2c* and *Myf5* did not affect *Myog* levels, contrary to *Myog* downregulation in Sol8 skeletal myoblasts overexpressing *Pitx2c*. The *Pax3* expression was not significantly altered in HL-1 cardiomyocytes that transiently overexpressed *Pitx2c*. The latter suggests that *Pax3* does not contribute to activation of *Myf5* expression in HL-1 cardiomyocytes, similarly to that occurring in the progenitor cells of postnatal skeletal muscle [56].

The *Pitx2*-binding sites in the *Myf5* promoter region are conserved among species, and two potential *Pitx2*-binding sites exist in the mouse *Myf5* promoter. It has been demonstrated that PITX2 physically associates with these binding sites and activates the *Myf5* promoter in limb- and extra-ocular muscle-derived precursor cell lines [57]. In addition, *Pitx2* potently stimulates the myogenic differentiation program in adult skeletal muscle satellite cells [58]. The results of our transfection assays raise the intriguing possibility that *Pitx2*-*Myf5* expression crosstalk can also be operative in cardiomyocytes. In this regard, expression of both *Pitx2* and *Myf5* was detected in cardiomyocyte-like (CML) cells generated from pluripotent stem cells of patients with DCM due to mutations in the cardiac troponin T gene [59]. *In-vitro* forced expression of sarcoplasmic reticulum Ca^2+^ ATPase (*Serca2a*) improved contractility function of CML cells that, in turn, was associated with downregulation of *Pitx2* and *Myf5* mRNA levels in *Serca2a*-transduced cells (see the data set available at NCBI through accession number GSE35108). On the other hand, and complementary to these results, ectopic overexpression of *Myf5* in the heart activates a skeletal muscle gene expression that results in progressive cardiomyopathy [60], [61]. The latter suggests that aberrant *Pitx2*-*Myf5* co-activation seen in our model of DHF may negatively impact on diastolic cardiac function.

Formally viewed, our findings suggest the involvement of *Pitx2c* in regulation of *Myf5* expression in the stressed myocardium. However, the expression of *Myf5* is modulated by a large number of endogenous signalling factors, each of which can have either positive or negative effects on *Myf5* expression, depending on cell type and physiological/pathological context [40], [62]. An inverse correlation in expression of *Pitx2c* (i.e., upregulation) and *Myf5* (i.e., downregulation) was detected in human samples of DCM and ICM (see Fig. 5). The discordance in *Myf5* expression in porcine versus human failing myocardium could be attributable to multiple factors, including differences in HF dysfunction phenotypes (diastolic versus systolic HF), stage and severity of HF (mid-advanced versus end-stage HF), age at which HF occurs (neonatal versus adult/aged heart), and medication (non-drug for DHF versus multidrug therapy for SHF). In addition, we observed a certain variability in *Pitx2c* and *Myf5* expression between DCM and ICM samples (see Fig. 5). At the molecular level, the difference in cardiac expression of inhibitors of skeletal myogenesis might also play a role. Myostatin, a negative regulator of myogenesis *and Myf5/MyoD* expression [63], is downregulated in our model of porcine DHF [64], while significantly upregulated in human HF due to DCM and ICM [65], supporting thus this hypothesis.

### Expression of the MRF transcriptional cassette in the heart

In the present study we demonstrated the expression of *Myf5* in porcine and human LV-myocardium at both transcript and protein levels via qRT-PCR and Western blotting, respectively (see Fig. 3 and 5). Surprisingly, our gene expression studies revealed that, in addition to the *Myf5* gene, a number of genes involved in regulation of skeletal myogenesis are also expressed in postnatal pig (*MyoD*, *Myog*, *Myf6/Mrf4, Pax3*) and adult human (*Myog*) myocardium. These data are consistent with detection of *MyoD* expression in postnatal mouse heart [66] as well as *MyoD*, *Myog* [67] and *Myf5* [68] transcripts in postnatal/adult rat ventricular myocardium.

Forced expression of MRFs, especially *MyoD* and *Myf5*, can covert different non-muscle cell types into skeletal myoblasts *in vitro* (reviewed in [69]. However, the ectopic misexpression of either *Myf5* or *MyoD* in the heart *in-vivo*, triggering skeletal muscle-specific gene expression, does not lead to terminal skeletal muscle differentiation [70], [71]. It is tempting to speculate that cardio-enriched miRs might suppress the expression level of MRFs, thus preventing skeletal myoblast differentiation in postnatal myocardium. In this sense, it was found that two miRs of miR669 family (miR669a and miR669q) inhibit skeletal myogenesis in postnatal cardiac progenitors by suppressing *MyoD* expression. Notably, miR669a expression is reduced in cardiac progenitors isolated from patients with cardiomyopathies due to myocardial infarction or pathological LV-hypertrophy [72]. In this context, it appears to be important to determinate miR networks regulated by *Pitx2c* in adult myocardium and their putative actions on MRF expression in the failing heart.

In the classical muscle molecular context, expression of the MRFs is sensitive to *Pitx2* gene dose. It could be foreseeable, for this reason, that *Pitx2c* upregulation in the failing myocardium in response to different pathological stimuli could contribute to MRF expression and thus impaired cardiac function. Our results provide previously unrecognized evidence that *Pitx2c* is reactivated in postnatal/adult heart at HF that in turn is associated with modulation of *Myf5* expression in failing myocardium. These findings emphasize the particular importance for future studies to define the *Pitx2* signalling pathways in principal cell types (cardiomyocytes, fibroblasts, smooth muscle and vascular endothelium cells) of postnatal myocardium.

## Supporting Information

Figure S1 Representative qRT-PCR amplification plots for *Pitx2c* transcripts in the piglet left ventricle. **A** - Newborn (NB, green) versus 30-day-old (30 d, blue) normal piglets. **B** - PBS-injected (PBS, blue) versus Dox-injected (Dox, red) 30-day-old animals. *Rpl19* - internal standard levels. Arrows - threshold cycle (C_T_). FT - fluorescent threshold. RFU - relative fluorescent units. Under experimental conditions used, each primer pair yielded a single peak of dissociation on the melting curve (**C**) and a single band with the expected size on PAGE gel post-stained with SYBR Green I (**D**).
(TIF)

Figure S2 Representative qRT-PCR amplification plots for *Myf5* and *Myog* transcripts in the left ventricle of PBS- versus Dox-injected piglets. **A** - PBS-injected (PBS, blue) versus Dox-injected (Dox, red) 30-day-old animals. *Rpl19* - internal standard levels. Arrows - threshold cycle (C_T_). FT - fluorescent threshold. RFU - relative fluorescent units. Under experimental conditions used, each primer pair yielded a single band with the expected size on PAGE gel post-stained with SYBR Green I (**B**).
(TIF)

Figure S3 Expression of *Myog*, *Foxj1* and *Pax3* genes in *Pitx2c*-transfected HL-1 and Sol8 cells. Overall relative levels of *Myog*, *Foxj1* and *Pax3* transcripts in HL-1 and Sol8 cells transfected with V5-tagged *Pitx2c* vector at different doses (100-400 ng). CT – empty-vector transfected cells. Shown are results of qRT-PCR analysis. Data from six replicates of each transfection were pooled and averaged. *p≤0.05.
(TIF)

Table S1 Baseline characteristics of neonatal piglets injected with Dox or PBS three weeks after injections.
(DOCX)

Table S2 Patient characteristics for samples employed qRT-PCR and Western blot analyses.
(DOCX)

Table S3 Primers used in this study.
(DOCX)

Table S4 Selective data set derived from the microarray database.
(DOCX)
