# Supplementary figures and images for: Pitx2c Is Reactivated in the Failing Myocardium and Stimulates Myf5 Expression in Cultured Cardiomyocytes
Source: PLoS One. 2014 Mar 4;9(3):e90561. doi: 10.1371/journal.pone.0090561 (PMC3942452; doi:10.1371/journal.pone.0090561)

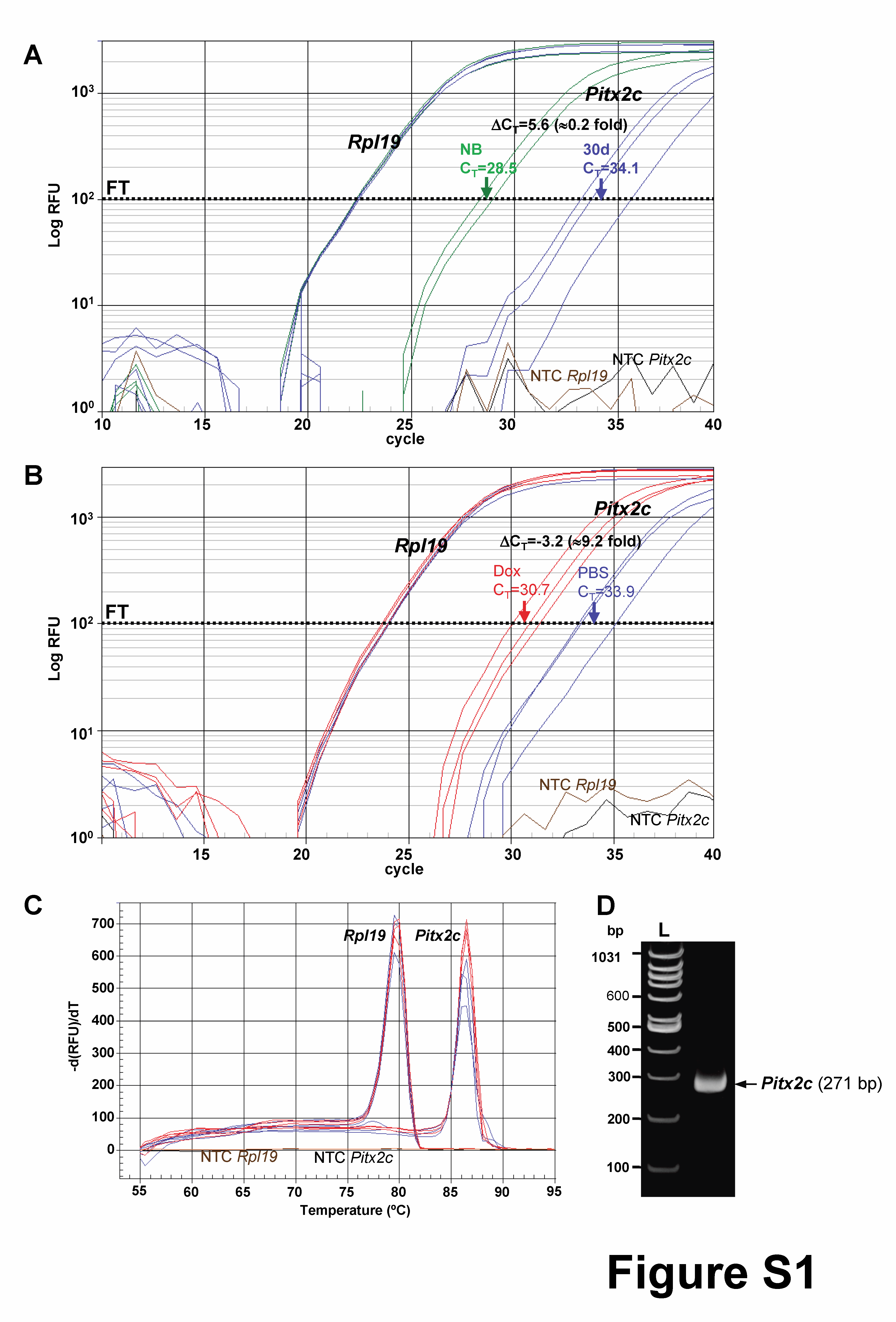

Supplement: Figure S1 — Representative qRT-PCR amplification plots for Pitx2c transcripts in the piglet left ventricle. A - Newborn (NB, green) versus 30-day-old (30 d, blue) normal piglets. B - PBS-injected (PBS, blue) versus Dox-injected (Dox, red) 30-day-old animals. Rpl19 - internal standard levels. Arrows - threshold cycle (CT). FT - fluorescent threshold. RFU - relative fluorescent units. Under experimental conditions used, each primer pair yielded a single peak of dissociation on the melting curve (C) and a single band with the expected size on PAGE gel post-stained with SYBR Green I (D). (TIF) [file pone.0090561.s001.tif]

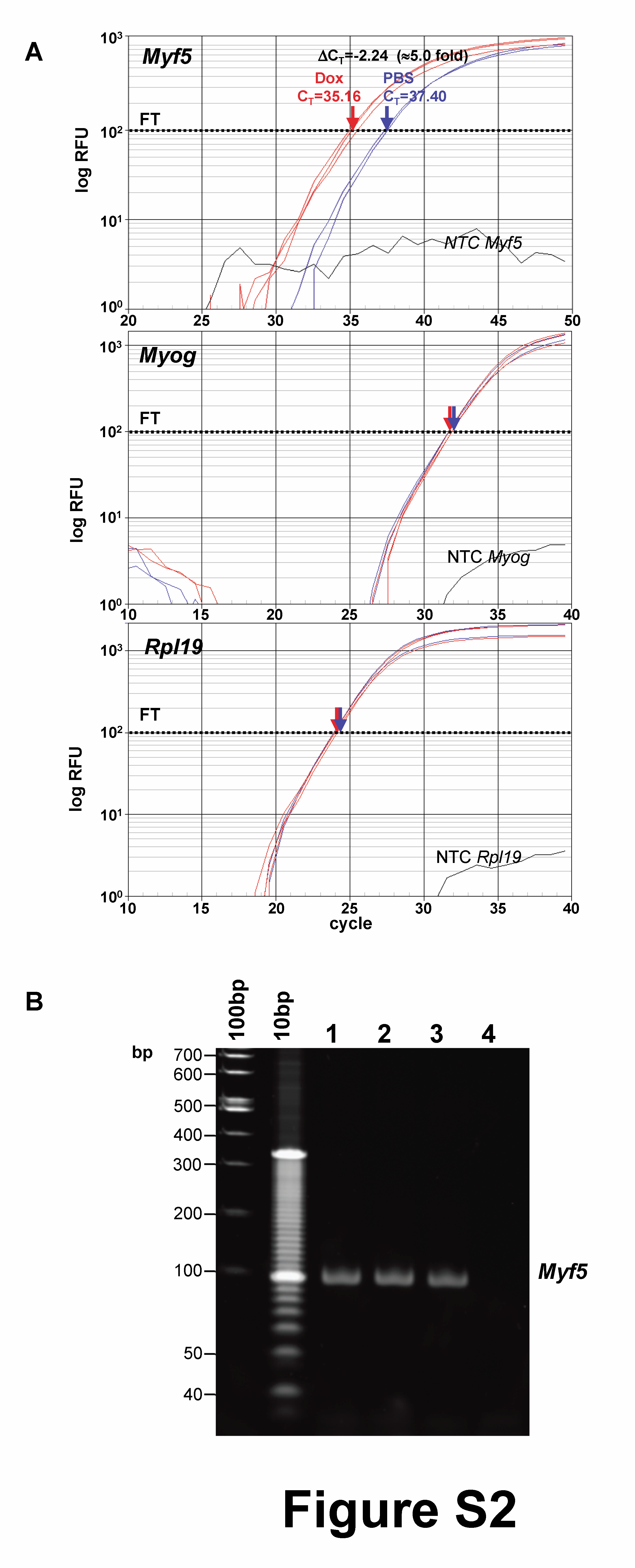

Supplement: Figure S2 — Representative qRT-PCR amplification plots for Myf5 and Myog transcripts in the left ventricle of PBS- versus Dox-injected piglets. A - PBS-injected (PBS, blue) versus Dox-injected (Dox, red) 30-day-old animals. Rpl19 - internal standard levels. Arrows - threshold cycle (CT). FT - fluorescent threshold. RFU - relative fluorescent units. Under experimental conditions used, each primer pair yielded a single band with the expected size on PAGE gel post-stained with SYBR Green I (B). (TIF) [file pone.0090561.s002.tif]

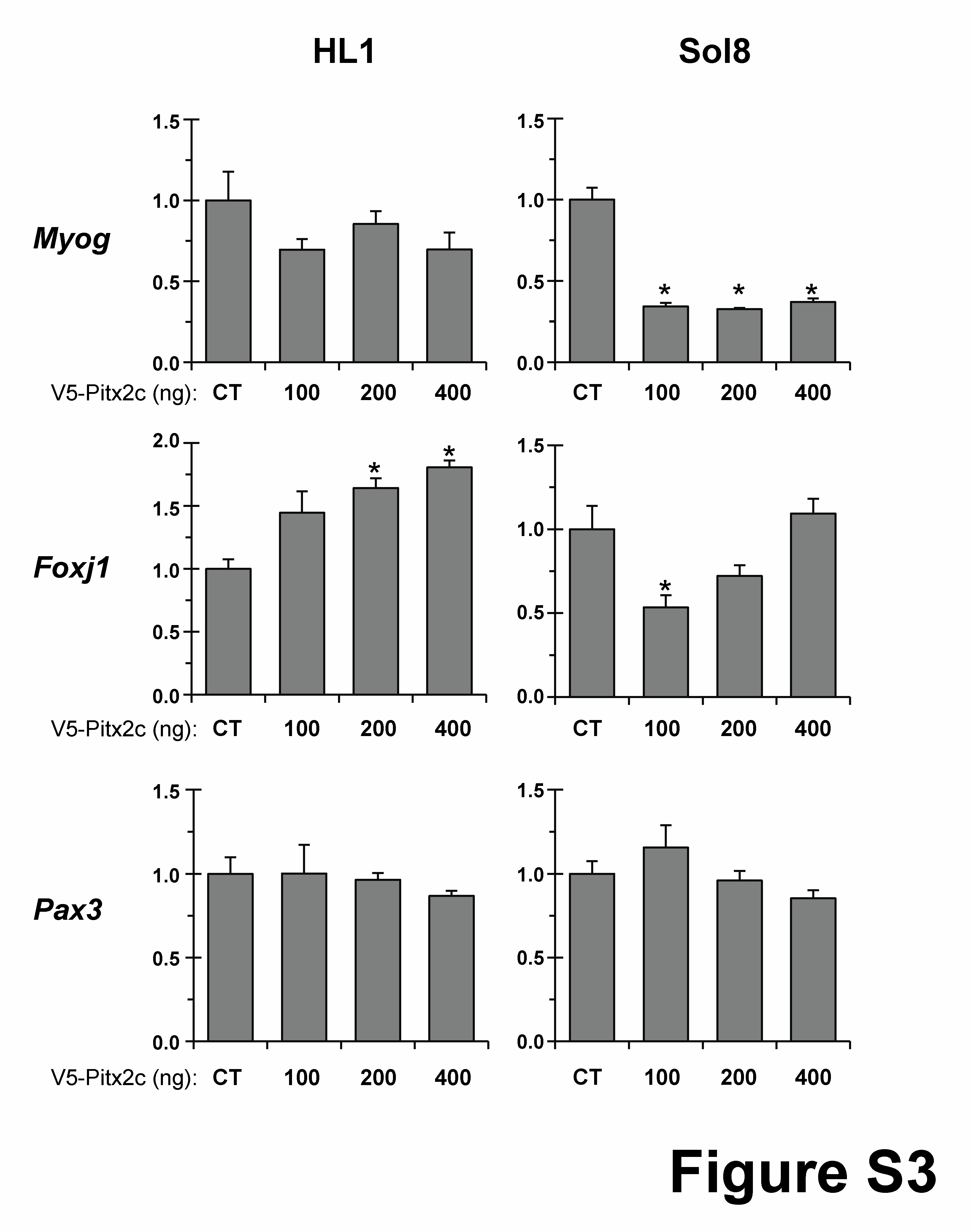

Supplement: Figure S3 — Expression of Myog, Foxj1 and Pax3 genes in Pitx2c-transfected HL-1 and Sol8 cells. Overall relative levels of Myog, Foxj1 and Pax3 transcripts in HL-1 and Sol8 cells transfected with V5-tagged Pitx2c vector at different doses (100-400 ng). CT – empty-vector transfected cells. Shown are results of qRT-PCR analysis. Data from six replicates of each transfection were pooled and averaged. *p≤0.05. (TIF) [file pone.0090561.s003.tif]
