# Supplementary material for: Pitx2c Is Reactivated in the Failing Myocardium and Stimulates Myf5 Expression in Cultured Cardiomyocytes
Source: PLoS One. 2014 Mar 4;9(3):e90561. doi: 10.1371/journal.pone.0090561 (PMC3942452; doi:10.1371/journal.pone.0090561)
Supplement: Table S1 — Baseline characteristics of neonatal piglets injected with Dox or PBS three weeks after injections. (DOCX) [file pone.0090561.s004.docx]

**Table S1. Baseline characteristics of neonatal piglets injected with Dox or PBS three weeks after injections**

| **Parameter** | **PBS, control** | **Dox, 2 mg/kg** |
| --- | --- | --- |
| Number of animals | 8 | 12 |
| Heart/body ratio, x1000 | 6.9±0.4 | 7.0±0.3 |
| Heart rhythm, beats/min | 125±7 | 110±5 |
| Low-voltage QRS, n | 0 | 7 |
| Ischemic T-wave at lead aVL, % | 0 | 100 |
| LV end-systolic pressure, mm Hg | 76.0±4.2 | 70.1±3.2 |
| LV end-diastolic pressure, mm Hg | 6.1±1.1 | 10.7±0.8* |
| Cardiac output, L/min/m2 | 3.4±0.4 | 3.2±0.6 |
| Extravascular lung water, ml/kg | 57.2±8.4 | 115.4±21.5* |
| LV nppb mRNA, fold change | 1 | 5.8±0.5 |
| LV ΔE2-nppb mRNA, fold change | 1 | 9.4±0.6 |
| Cardiac arrest, n | 0 | 2 |

Nppb – natriuretic peptide precursor B; ΔE2-nppb – exon 2 skipped
