# Supplementary material for: Pitx2c Is Reactivated in the Failing Myocardium and Stimulates Myf5 Expression in Cultured Cardiomyocytes
Source: PLoS One. 2014 Mar 4;9(3):e90561. doi: 10.1371/journal.pone.0090561 (PMC3942452; doi:10.1371/journal.pone.0090561)
Supplement: Table S2 — Patient characteristics for samples employed qRT-PCR and Western blot analyses. (DOCX) [file pone.0090561.s005.docx]

**Table S2. Patient characteristics for samples employed q-RT-PCR and Western blot analyses**

| **Patient** | **Tissue** | **Age, years** | **Sex** | **Diagnosis** | **LVEF, %** | **NYHA, I-IV** | **Last medication** |
| --- | --- | --- | --- | --- | --- | --- | --- |
| H28R | LV | 61 | Male | isch-DCM | 10 | IV | En, D |
| H32R | LV | 47 | Male | id-DCM | 15 | IV | En, D |
| H163R | LV | 18 | Female | id-DCM | 22 | IV | D, En, F, AC |
| H49R | LV | 67 | Female | id-DCM | 25 | IV | F, D, H |
| H10R | LV | 65 | Male | id-DCM | 16 | IV | En, D |
| HH21R | LV | 64 | Female | id-DCM | 10 | III | F, A-II ,B, AC |
| HH26R | LV | 51 | Male | isch-DCM | 19 | IV | AC, F, A-II ,D ,B |
| HH27R | LV | 65 | Male | isch-DCM | 17 | III | B, Am ,D |
| HH28R | LV | 54 | Female | isch-DCM | 20 | III | D, B, A |
| H58R | LV | 64 | Male | isch-DCM | 36 | IV | H, F, Am |
| H11R | LV | 58 | Male | isch-DCM | 22 | IV | H |
| H46R | LV | 62 | Male | isch-DCM | 16 | IV | En, F, D |
| H55R | LV | 59 | Male | isch-DCM | 20 | IV | F, AC, D, En |
| F242R | LV | 23 | Male | id-DCM | 16 | IV | F, H |
| F221R | LV | 53 | Male | id-DCM | 14 | IV | F, En, D, H |
| F367R | LV | 46 | Male | id-DCM | 23 | III | En, D, F |
| F391R | LV | 60 | Male | id-DCM | 9 | III | D, F, En, Am, B |
| H8D | LV | 30 | Male | FA | ND | ND | ND |
| H21D | LV | 20 | Male | FA | ND | ND | ND |
| H22D | LV | 43 | Male | FIH | ND | ND | ND |
| H33D | LV | 32 | Male | CT | ND | ND | ND |
| H38D | LV | 42 | Male | CT | ND | ND | ND |
| H41D | LV | 30 | Male | CT | ND | ND | ND |
| H47D | LV | 26 | Male | CT | ND | ND | ND |
| HH11D | LV | 44 | Female | FA | ND | ND | ND |
| HH12D | LV | 59 | Female | BTD | 60 | ND | ND |
| HH14D | LV | 56 | Female | FIH | ND | ND | ND |
| HH15D | LV | 50 | Male | CI | ND | ND | ND |

LVEF - left ventricular ejection fraction; NYHA, I-IV - heart failure stages according to the New York Heart Association classification; ish-DCM - ischemic dilated cardiomyopathy; id-DCM - idiopathic DCM; FA - fatal accident; FIH - fatal intracranial hemorrhage; CT - cerebral trauma; BTD - brain tumor death; CI - cerebral infarct; A-II - angiotensin receptor antaginists; B - beta-blokers; D - digitalis; H - heparin; F-furosemid; Am - amiodarone; AC - anticoagulants; En - enapril; A - ACE-inhibitors; ND - data not available.
