# Supplementary material for: Pitx2c Is Reactivated in the Failing Myocardium and Stimulates Myf5 Expression in Cultured Cardiomyocytes
Source: PLoS One. 2014 Mar 4;9(3):e90561. doi: 10.1371/journal.pone.0090561 (PMC3942452; doi:10.1371/journal.pone.0090561)
Supplement: Table S3 — Primers used in this study. (DOCX) [file pone.0090561.s006.docx]

| **Primer** | **F/R** | **Target** | **Species** | **Sequence (5´-3´)** | **Product length** | **Application** |
| --- | --- | --- | --- | --- | --- | --- |
| 369 | F | *Pitx2c* | P | tgaaaggcccgctgcacttg |  |  |
| 354 | R | *Pitx2* | P | gaggagttggcggtgcgtaag | 369-354: 825 bp (Pitx2c) | RT-PCR |
| 373 | F | *Pitx2b* | P | gcagccagcagcaagctcttc | 373-354: 670 bp (Pitx2b) | RT-PCR |
| 372 | F | *Pitx2a* | P | cggcgtgtgtgcaattagagaaagac | 372-354: 642 bp (Pitx2a) | RT-PCR |
| 350 | F | *Pitx2* | P | catgtccacgcgtgaagaaatc |  |  |
| 353 | R | *Pitx2* | P | tgcggcccaaagccattcttg | 350-353: 144 bp (Pitx2) | qPCR |
| 376 | F | *Pitx2c* | P | gcacaccatctctgacacctccag |  |  |
| 352 | R | *Pitx2* | P | tttggcccggcgattcttgaac | 376-352: 271 bp (Pitx2c) | qPCR |
| 391 | F | *Pitx2c* | H | gcacaccatctccgacacctccag |  |  |
| 392 | R | *Pitx2c* | H | tgccgcttcttcttagacgggtcctc | 391-392: 108 bp | qPCR |
| 410 | F | *Myf5* | P | cagcagttttgacagtatctactgtcc |  |  |
| 411 | R | *Myf5* | P | atccgatccactatgctggataagc | 410-411: 105 bp | qPCR |
| 408 | F | *Myf5* | H | gcagtacttttgacagcatctactgtcct |  |  |
| 409 | R | *Myf5* | H | actatgttggataagcaatccaagctgg | 408-409: 97 bp | qPCR |
| 404 | F | *Myog* | P/H | cacagcgcctcctgcagtc |  |  |
| 407 | R | *Myog* | P/H | catggtttcatctgggaaggccacag | 404-407: 174 bp | qPCR |
| 420 | F | *Foxj1* | P | cagcaaggccaccaagatcac |  |  |
| 421 | R | *Foxj1* | P | agcacttgttcagagacaggttgtg | 420-421:128 bp | qPCR |
| 416 | F | *Pax3* | P | accttcacagcagagcagcttg |  |  |
| 417 | R | *Pax3* | P | atggttgaaagccatcagttgattgg | 416-417:192 bp | qPCR |
| 64 | F | *Rl19* | P/H | ctgctcagaagataccgtgaat |  |  |
| 206 | R | *Rpl19* | P | gcttgtggatgtgctccatga | 64-206:121 bp | qPCR |
| 207 | R | *Rpl19* | H | gcttgtggatgtgttccatga | 64-207:121 bp | qPCR |
| MmPitx2cF | F | *pitx2c* | M | cctcacccttctgtcaccat |  |  |
| MmPitx2cR | R | *pitx2c* | M | gcccacatcctcattctttc | 179 bp | qPCR |
| MmFoxj1F | F | *Foxj1* | M | acacgtgaagccaccctact |  |  |
| MmFoxj1R | R | *Foxj1* | M | tgttcaaggacaggttgtgg | 176 bp | qPCR |
| MmMyf5F | F | *Myf5* | M | tgagggaacaggtggagaac |  |  |
| MmMyf5R | R | *Myf5* | M | agctggacacggagctttta | 198 bp | qPCR |
| MmPax3F | F | *Pax3* | M | gccaatcaactgatggcttt |  |  |
| MmPax3R | R | *Pax3* | M | gtacagtgctcggaggaagc | 181 bp | qPCR |
| MmMyogF | F | *Myog* | M | ctacaggccttgctcagctc |  |  |
| MmMyogR | R | *Myog* | M | acgatggacgtaagggagtg | 221 bp | qPCR |
| MmGapdhF | F | *Gapdh* | M | ggcattgctctcaatgacaa |  |  |
| MmGapdhR | R | *Gapdh* | M | ggcattgctctcaatgacaa | 200 bp | qPCR |
| MmGusbF | F | *Gusb* | M | acgcatcagaagccgattat |  |  |
| MmGusbR | R | *Gusb* | M | actctcagcggtgactggtt | 212 bp | qPCR |

**Table S3. Primers used in this study**

F/R – forward/reverse primers; P – pig; H – human: M – mouse.
