# Supplementary material for: Pitx2c Is Reactivated in the Failing Myocardium and Stimulates Myf5 Expression in Cultured Cardiomyocytes
Source: PLoS One. 2014 Mar 4;9(3):e90561. doi: 10.1371/journal.pone.0090561 (PMC3942452; doi:10.1371/journal.pone.0090561)
Supplement: Table S4 — Selective data set derived from the microarray database. (DOCX) [file pone.0090561.s007.docx]

**Table S4. Selective data set derived from the microarray database (DHF failing vs. non-failing myocardium.**

| **Probe Set ID** | **Gene**  **symbol** | **Gene name** | **Fold Change** | **P value** |
| --- | --- | --- | --- | --- |
| **Skeletal myogenic pathway** | | | | |
| Ssc.27397.1.A1_at | MYF5 | myogenic factor 5 | 5.15 | 0.12 |
| Ssc.27600.1.S1_at | PAX3 | paired box 3 | 3.39 | 0.21 |
| Ssc.20571.2.S1_at | TPM2 | tropomyosin 2 (beta) | 1.97 | 0.24 |
| Ssc.16626.1.S1_at | MYF6 | myogenic factor 6 (herculin) | 1.69 | 0.15 |
| SscAffx.10.1.S1_at | MYOD1 | myogenic differentiation 1 | 1.44 | 0.41 |
| Ssc.15909.1.S1_at | MYH4 | myosin, heavy chain 4, skeletal muscle | 1.40 | 0.71 |
| Ssc.13874.2.A1_s_at | ACTA1 | actin, alpha 1, skeletal muscle | 1.26 | 0.09 |
| Ssc.7535.1.S1_at | TNNT1 | troponin T type 1 (skeletal, slow) | 1.07 | 0.90 |
| Ssc.24147.1.S1_at | TNNT3 | troponin T type 3 (skeletal, fast) | 1.04 | 0.85 |
| Ssc.9096.2.A1_at | MDFIC | MyoD family inhibitor domain containing | -1.88 | 0.03 |
| Ssc.14459.1.S1_a_at | MYL1 | myosin, light chain 1, alkali; skeletal, fast | -4.58 | 0.04 |
| **Transforming growth factor beta pathway** | | | | |
| Ssc.4253.1.S1_at | TGFBR3 | transforming growth factor, beta receptor III | 6.20 | 0.17 |
| Ssc.15913.1.S1_at | SMAD3 | SMAD family member 3 | 2.00 | 0.09 |
| Ssc.92.1.A1_at | TGFB2 | transforming growth factor, beta 2 | 1.60 | 0.07 |
| Ssc.24152.1.A1_at | SMAD6 | SMAD family member 6 | 1.09 | 0.73 |
| Ssc.120.1.S1_at | ENG | endoglin | 1.04 | 0.84 |
| Ssc.76.3.S1_a_at | TGFB1 | transforming growth factor, beta 1 | -1.05 | 0.83 |
| Ssc.23794.1.S1_at | TGFBR1 | transforming growth factor, beta receptor 1 | -1.09 | 0.82 |
| Ssc.249.1.S1_at | SMAD4 | SMAD family member 4 | -1.26 | 0.22 |
| Ssc.11757.1.S1_at | SMAD1 | SMAD family member 1 | -1.57 | 0.04 |
| **Wnt/β-catenin pathway** | | | | |
| Ssc.14176.1.A1_s_at | GSK3B | glycogen synthase kinase 3 beta | 2.13 | 0.23 |
| Ssc.27552.1.A1_at | FZD9 | frizzled family receptor 9 | 2.08 | 0.23 |
| Ssc.10817.2.A1_at | WNT10B | wingless-type MMTV integration site family, member 10B | 1.88 | 0.39 |
| Ssc.20193.1.S1_at | FZD3 | frizzled family receptor 3 | -1.11 | 0.76 |
| Ssc.27747.1.S1_at | WNT2B | wingless-type MMTV integration site family, member 2B | -1.17 | 0.65 |
| Ssc.14003.1.S1_a_at | CTNNB1 | catenin (cadherin-associated protein), beta 1, 88kDa | -1.33 | 0.12 |
| Ssc.10434.1.S1_at | FZD8 | frizzled family receptor 8 | -1.87 | 0.09 |
| Ssc.26237.1.A1_at | WNT6 | wingless-type MMTV integration site family, member 6 | -2.16 | 0.10 |
| Ssc.24595.1.S1_at | WNT5B | wingless-type MMTV integration site family, member 5B | -4.28 | 0.02 |
| Ssc.19566.1.S1_at | SFRP4 | secreted frizzled-related protein 4 | -5.59 | 0.04 |
